# Leucettamols, Bifunctionalized Marine Sphingoids, Act as Modulators of TRPA1 and TRPM8 Channels 

**DOI:** 10.3390/md10112435

**Published:** 2012-11-02

**Authors:** Giuseppina Chianese, Ernesto Fattorusso, Masteria Yunovilsa Putra, Barbara Calcinai, Giorgio Bavestrello, Aniello Schiano Moriello, Luciano De Petrocellis, Vincenzo Di Marzo, Orazio Taglialatela-Scafati

**Affiliations:** 1 Department of Natural Compounds Chemistry, University of Naples Federico II, Via Montesano 49, 80131 Napoli, Italy; Email: g.chianese@unina.it (G.C.); mas_ter52@yahoo.co.id (M.Y.P.); 2 Department of Marine Science, Polytechnic University of Marche, Via Brecce Bianche, 60131 Ancona, Italy; Email: b.calcinai@univpm.it; 3 Department for the Study of the Territory and its Resources, University of Genoa, Corso Europa 26, 16132, Genova, Italy; Email: g.bavestrello@univpm.it; 4 Endocannabinoid Research Group, Istituto di Cibernetica-CNR, Via Campi Flegrei 34, 80078 Pozzuoli (Naples), Italy; Email: a.schianomoriello@cib.na.cnr.it (A.S.M.); l.depetrocellis@cib.na.cnr.it (L.D.P.); 5 Endocannabinoid Research Group, Istituto di Chimica Biomolecolare-CNR, Via Campi Flegrei 34, 80078 Pozzuoli (Naples), Italy; Email: vdimarzo@icmib.na.cnr.it

**Keywords:** leucettamols, TRP receptors, pain modulation

## Abstract

Leucettamols, bifunctionalized sphingoid-like compounds obtained from a marine sponge *Leucetta *sp., act as non-electrophilic activators of the TRPA1 channel and potent inhibitors of the icilin-mediated activation of the TRPM8 channel, while they are inactive on CB_1_, CB_2_ and TRPV1 receptors. Leucettamols represent the first compounds of marine origin to target TRPA1 and the first class of natural products to inhibit TRPM8 channels. The preparation of a small series of semi-synthetic derivatives revealed interesting details on the structure-activity relationships within this new chemotype of simple acyclic TRP modulators.

## 1. Introduction

The transient receptor potential (TRP) proteins are a superfamily of non selective cation channels (permeable to both monovalent and divalent cations) characterized by six transmembrane domains, a pore forming loop, and COOH/NH_2_ termini located in the cytosol [1]. About thirty mammalian TRP channels have been identified and grouped into six main subfamilies: TRPC (canonical), TRPA (ankyrin), TRPV (vanilloid), TRPM (melastatin), TRPP (polycystin), TRPML (mucolipin) [2]. TRP channels are considered to be ubiquitous in the human organism and they are expressed practically in almost every tissue and cell type where they regulate several cell functions [3]. For example, some TRP channels have been demonstrated to be strongly involved in the mediation of pain, taste, hot or cold sensations and they can be considered as cellular sensors for external stimuli [4]. However, fifteen years after the cloning of the first TRP channel (TRPV1) [5], the detailed physiological role of these channels, as well as their involvement in human pathological conditions, is still yet to be fully disclosed. Only a few channelopathies have been identified [6] but there is evidence that a dysregulation in the TRP activity is implicated in the pathogenesis of many other diseases, thus TRP agonists/antagonists could provide interesting pharmacological options in several pathologies [7]. 

In view of their involvement in the detection of pain-evoking noxious stimuli, TRPA1 and TRPM8, along with TRPV1, are probably the most intensely investigated TRP channels. TRPA1 seems to play a great role in noxious cold perception and has been implicated in neuropathic and inflammatory pain [8]; consequently, it is an emerging target for novel analgesic and anti-inflammatory agents. TRPM8 is a neuronal sensor activated by innocuous cool and noxious cold temperatures [9] and plays a role in cold and mechanical allodynia arising from traumatic neuropathy, in conjunction with TRPA1 [10]. Recent studies have also shown that TRPM8 is markedly expressed in visceral organs, predominantly in prostate and liver [11] and, more importantly, it is highly upregulated in prostate cancer cells [12]. Some Authors have hypothesized that the TRPM8-mediated Ca^2+^ efflux may play a role in prostate tumour cell proliferation [13]. Thus, antagonism of TRPM8 activation could represent a novel and potentially useful approach for the treatment of painful conditions, such as cold allodynia and cold hyperalgesia, for which there continues to be a high unmet medical need, but it is also an innovative strategy for the treatment of urological disorder, especially prostate cancer, a major health problem in adult males.

In view of their incredible potential for biomedical and nutritional research, it is not surprising that recent years have witnessed an intense research activity aimed at finding specific ligands for the different TRP channels. Endogenous ligands such as arachidonic acid and linolenic acid derivatives activate TRP channels [14], while other lipid mediators, such as diacylglycerols, have been described to open some mammalian TRPC channels [15]. Natural products play a prominent role as TRP ligands [16] and many TRP channels are activated/antagonized by the so-called “chemestetical” secondary metabolites found in some natural sources, especially spices: for example allyl isothiocyanate (horseradish and wasabi), diallylthiosulfinate (garlic) and cinnamaldehyde (cinnamon) are activators of the sensory ion channel TRPA1 [17], menthol (mint) and eucalyptol (eucalyptus) activate the cold receptor TRPM8 [18], vanilloids as capsacin (chilli pepper) but also cannabidiol (hemp) stimulate TRPV1 [19]. In this latter case, it is interesting to note the existence of a close functional cross-talk between TRPV1 and cannabinoid (CB) receptors; indeed, TRPV1 and the related channel TRPV4 have been shown to be opened by the endocannabinoid anandamide, which also antagonizes TRPM8 channels [20]. Natural product ligands continue to be investigated as valuable leads for the deorphanisation of TRP ion channels since new TRP modulators are strongly needed both as tools for further biological studies on these multifunctional channels and as viable candidates for drug discovery.

**Scheme 1:**
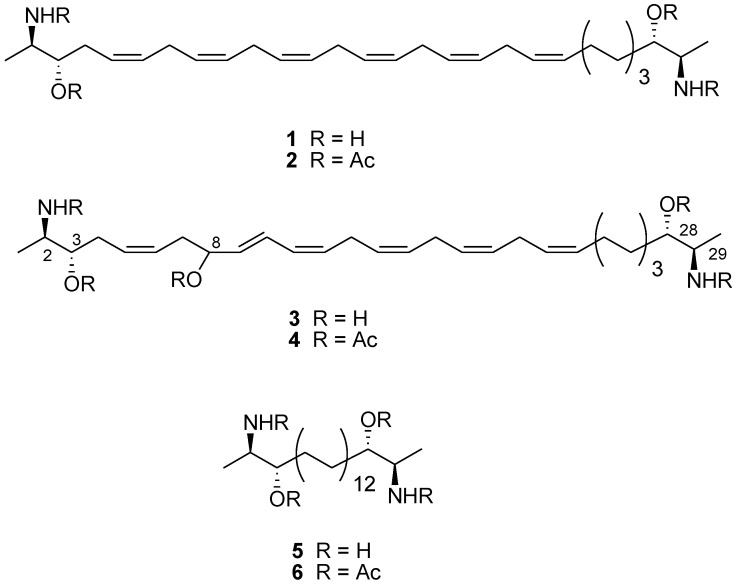
Chemical structures of leucettamols A (**1**) and B (**3**) and of their semi-synthetic analogues.

We have been actively working in this field and have recently reported the identification of some TRP [21,22,23,24] and CB [25] ligands from natural sources, e.g., zerumbone and curcumin identified as TRPA1 ligands [23]. In this paper we now report that leucettamols A (**1**) and B (**3**) (Scheme 1), two-headed sphingoid-like compounds obtained from the marine sponge *Leucetta *sp., act as potent modulators of TRPA1 and TRPM8 channels, while they do not show any activity on CB and TRPV1 receptors. Some preliminary structure-activity relationships in this new chemotype of simple acyclic TRP modulators, deduced through the preparation of a small series of semi-synthetic derivatives, will be also discussed here. 

## 2. Results and Discussion

### 2.1. Chemistry

In the frame of our ongoing investigation of Indonesian invertebrates for bioactive secondary metabolites [26,27,28], we had the opportunity to analyze a specimen of the sponge *Leucetta* sp. (family Leucettidae, order Clathrinida), collected along the Island of Siladen, in the Bunaken Marine Park of Manado (North Sulawesi, Indonesia). The sponge (310 g wet weight) was extracted with MeOH and CHCl_3_ at room temperature and the combined organic extracts (8.6 g) were partitioned between H_2_O and EtOAc to give an acetate extract of 0.45 g, while the water phase was further partitioned against *n*-BuOH, thus affording a butanol extract (1.2 g). This was chromatographed by MPLC (medium pressure liquid chromatography) over silica gel eluting with a gradient system of increasing polarity from EtOAc to MeOH, and the obtained fractions were further purified by RP18 HPLC affording leucettamols A (**1**, 452.0 mg) and B (**3**, 45.5 mg) in the pure state. The structures of these compounds were assigned through comparison of their spectral data with those reported in the literature [29].

Leucettamols are C_30_ bifunctionalized sphingoid-like compounds first reported from the Micronesian *Leucetta microrhaphis* and then demonstrated to moderately inhibit the interaction of the ubiquitin enzyme Ubc13 and Uev1A, thus up-regulating the activity of the tumour suppressor p53 protein and qualifying as a possible anti-cancer leads [30]. In a recent paper, Molinski *et al.* have determined the absolute configurations at the four stereogenic centers of leucettamol A (**1**) as 2*R*, 3*S*, 28*S*, 29*R* by analysis of deconvoluted exciton coupled circular dichroism [31]. In the same paper, the authors also postulated that the co-occurring leucettamol B (**3**) should possess the same absolute configuration at the parallel stereogenic carbons, but they left undetermined the absolute configuration at C-8, suggesting an artifact origin for this stereocenter. In this regard, it should be considered that the organic extract obtained from *Leucetta* sp. did not appear to contain any regioisomer of leucettamol B, thus excluding “random” pattern of oxygenation. We tried to complete the stereostructural elucidation of **3** through application of the Mosher’s method for secondary alcohols but, unfortunately, in the reaction conditions leucettamol B experienced a severe degradation which prevented any unambiguous stereochemical determination. 

Standard acetylation (pyridine/acetic anhydride) of leucettamols A (**1**) and B (**3**) yielded the tetra-acetylated **2** and the penta-acetylated **4**, respectively (Scheme 1). Compound **1** (34.0 mg) was also treated with palladium-charcoal in EtOH (6 mL) under a hydrogen atmosphere for 18 h to afford, after filtration of the catalyst and HPLC purification, the saturated compound **5** (36.5 mg, 100%). An aliquot of **5** (8.0 mg) was then acetylated to give **6** in quantitative yields (Scheme 1). 

### 2.2. Activity at CB Receptors and TRP Channels

Inspired by a certain structural resemblance of leucettamols with anandamide (*N*-arachidonoylethanolamide), one of the main endogenous agonists of cannabinoid receptors, we decided to evaluate the affinity of **1**–**6** on these end-points. Leucettamols A and B and their semisynthetic analogues did not exhibit significant affinity for both CB_1_ and CB_2_ (*K*_i_ > 10 μM, data not shown), with no difference between non-acetylated and acetylated analogues. Then, considering that anadamide also behaves as modulators toward some TRP channels (*i.e.*, TRPV1 and TRPM8) [20], compounds **1**–**6** were also tested on the three most intensely investigated TRP channels (TRPV1, TRPA1, and TRPM8), which play an important role in the detection of painful stimuli. Within the six tested compounds only leucettamol B (**3**) showed a moderate (EC_50_ = 4.8 μM, data not shown) elevation of intracellular Ca^2+^ in HEK-293 cells stably transfected with the human recombinant TRPV1 channel with a very low efficacy (14.96 ± 0.03% of 4 μM ionomycin at 10 μM while at the same concentration, capsaicin was 78.6 ± 2.4%). In contrast, compounds **1**–**6** showed a potent activating activity on the rat TRPA1 channel (Table 1). Using a fluorometric test, we observed that rat TRPA1-HEK293 cells exhibited an increase in intracellular [Ca^2+^]_i_ upon application of **1**–**6**. All compounds showed a potency in the range 2.5–10 μM with the completely saturated analogue **5 **being the most potent compound of the series (EC_50_ = 2.6 μM), while its acetylated analogue **6** was the less potent compound with an about four times reduction of the activity. The activity of the compounds was normalised to the maximum intracellular Ca^2+^ elevation generated by application of allylisothiocyanate (AITC) 100 μM. Almost all the compounds (with exception of **5**) were able to reach the maximal response of AITC, which was markedly overcome by peracetylated leucettamol A (**2**). 

**Table 1:** Efficacy and potency of compounds **1**–**6** on TRPA1 channel and inhibitory (Inh.) effect on TRPM8 channel.

	Potency EC_50_ TRPA1 (μM)	Efficacy TRPA1 (% AITC 100 μM)	IC_50_ Inh. TRPA1 μM (AITC 100 μM)	IC_50_ Inh. TRPM8 μM (icilin 0.25 μM)
Leucettamol A (**1**)	3.7 ± 1.7	101.9 ± 12.4	4.7 ± 0.2	6.5 ± 0.3
**2**	9.4 ± 2.2	139.2 ± 7.5	11.6 ± 2.3	44.6 ± 10.1
Leucettamol B (**3**)	5.9 ± 1.9	103.3 ± 10.6	4.7 ± 0.9	6.4 ± 1.0
**4**	3.5 ± 2.5	109.6 ± 21.0	32.6 ± 4.8	65.7 ± 8.6
**5**	2.6 ± 0.1	69.2 ± 0.1	6.5 ± 0.4	6.5 ± 0.3
**6**	9.7 ± 3.3	100.0 ± 9.7	12.7 ± 0.8	29.0 ± 0.6

**Figure 1:**
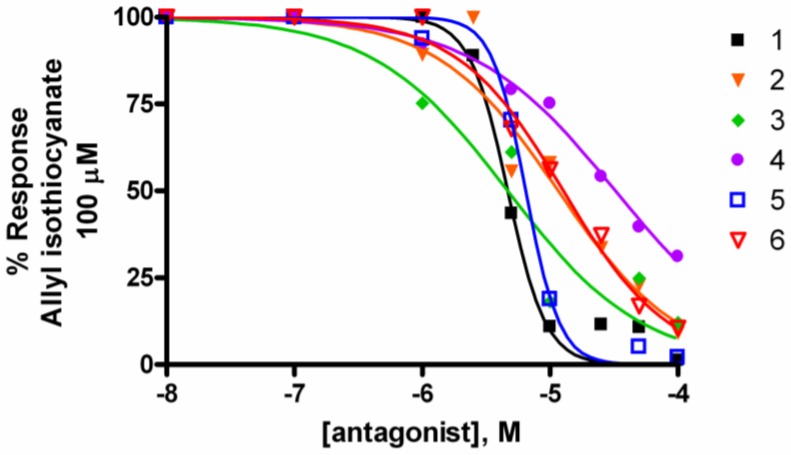
Desensitization of TRPA1-HEK-293 as a result of 5-min pre-incubation of cells with **1**–**6**. These compounds prevented the elevation of [Ca^2+^]_i_ induced by AITC 100 μM in a concentration-dependent manner. Responses were measured as peak increases in fluorescence and expressed as percentages of the uninhibited response.

**Figure 2:**
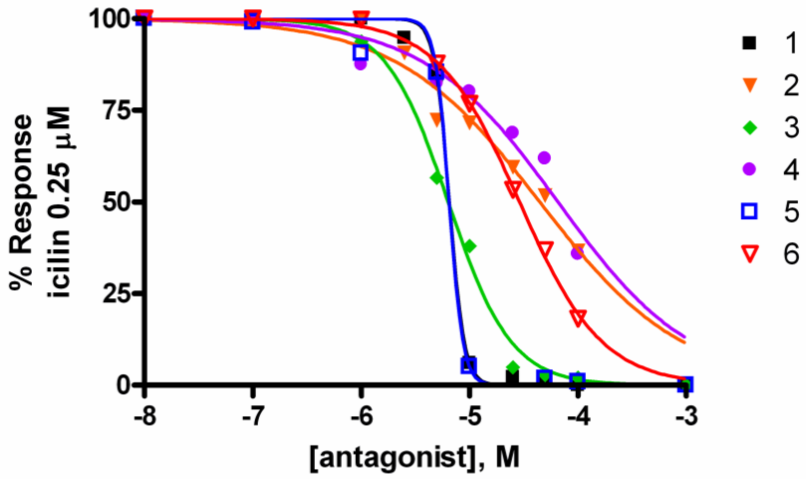
Inhibition of TRPM8-HEK-293 as a result of 5-min pre-incubation of cells with **1**–**6**. These compounds prevented the elevation of [Ca^2+^]_i_ induced by icilin 0.25 μM in a concentration-dependent manner. Responses were measured as peak increases in fluorescence and expressed as percentages of the uninhibited response.

As potent TRPA1 activators, a 5-min preincubation of TRPA1-HEK-293 cells with **1**–**6** desensitized the channel and prevented the elevation of [Ca^2+^]_i_ induced by AITC in TRPA1-HEK-293 cells (Table 1 and Figure 1). Peracetylated leucettamol B (**4**) was the less potent analogue at desensitizing the channel. When tested on TRPM8-HEK-293 cells, compounds **1**–**6** showed no activating activity up to 100 μM, but leucettamols A and B and the saturated analogue **5**, pre-incubated (5 min) with the same cells, proved to be potent inhibitors of the activation of TRPM8 by icilin (0.25 μM). All the acetylated analogues (**2**, **4**, **6**) were markedly less active (Table 1 and Figure 2). 

The activity of leucettamol A (**1**) is practically identical to that of leucettamol B (**3**) on both TRPA1 and TRPM8 channels, indicating that the integrity of the double bond system is not required for activity. Accordingly, the completely saturated analogue **5**, whose activity at TRPM8 is almost identical to that of its parent compound **1**, while it exhibited only a 30% reduction of efficacy at TRPA1. Anandamide and NADA have been reported to antagonize TRPM8 [32] but the activity of compound **5 **demonstrates that the double bond system is likely not required for this activity. Compounds **2**, **4**, and **6** revealed that the effect of acetylation can be considered moderate as for interaction with TRPA1, since the potency of the acetylated analogues experiences an average reduction by one half, but the efficacy of these compounds is practically unchanged and the peracetylated leucettamol (**2**) is the most efficacious compound of the series. In contrast, the effect of acetylation on TRPM8 inhibition is more dramatic and all the peracetylated compounds are 5–10 times less active than their parent compounds.

Isothiocyanates, sulfinates and unsaturated aldehydes (as acrolein or cynnamaldehyde) are the archetypal activators of TRPA1. These compounds, as most of the known TRPA1 agonists, are believed to act via the formation of a reversible covalent bond with the thiol (cysteine) residues present in the ankyrin domain, as a result of a Michael-type reaction of their electrophilic carbon or sulfur atoms. In this regard, we have recently reported a simple NMR assay to identify and discriminate among reversible and irreversible thiol sinks [23] and those phytochemicals reacting efficiently with the model compound cysteamine proved to be also able to link and activate the TRPA1 channel [23]. Interestingly, leucettamols are new members of the small class of non-electrophilic agonists of TRPA1 and their activity in the low micromolar range is also remarkable.

The effect of leucettamols on TRPM8 channels is even more interesting. Indeed, while many agonists of TRPM8 have been described, there are very few channel antagonists reported in the literature, and among those reported, many also act on other somatosensory-related ion channels, such as TRPV1, suggesting a conserved mechanism amongst these channels. The reported TRPM8 antagonists belong to structurally diverse scaffolds, as menthylamides [33] and piperidines [34], but all these compounds are of synthetic origin and, thus, leucettamols can be indicated as the first class of natural products exhibiting activity as TRPM8 inhibitors. Very recently, some natural products have been demonstrated to inhibit different TRPM channels, as waixenicin A (TRPM7) [35] and quinine (TRPM7 [36] and TRPM5 [37]).

An interesting question stimulated by our discovery concerns the role of leucettamols in the producing organism. It should be noted that these compounds are major secondary metabolites of a sponge *Leucetta *sp. (about 6% of the organic extract) and, most likely, they have an important ecological role for the survival of this organism. Our discovery that leucettamols interact with TRP channels could provide a clue to shed light on this point, since they could play a defensive role against predators through production of pro-nociceptive actions on sensory neurons. This hypothesis is in line with the evidence that cnidarian venom causes erythema, burning pain, hypersensitivity and inflammation, through desensitization of TRPV1 both in humans and fishes [38]. Similarly, the polyether toxins gambierol and brevetoxin, which are believed to underlie ciguatera fish poisoning and neurotoxic shellfish poisoning, increase currents through pre-activated TRPV1 channels [39].

## 3. Experimental Section

### 3.1. General Experimental Procedures

Optical rotations (CHCl_3_) were measured at 589 nm on a P2000 Jasco polarimeter using a 10 cm microcell. ^1^H (500 MHz) and ^13^C (125 MHz) NMR spectra were measured on a Varian INOVA spectrometer. Chemical shifts were referenced to the residual solvent signal (CDCl_3_: δ_H_ 7.26, δ_C_ 77.0). Homonuclear ^1^H connectivities were determined by the COSY experiment; one-bond heteronuclear ^1^H-^13^C connectivities by the HSQC experiment; two- and three-bond ^1^H-^13^C connectivities by gradient-HMBC experiments optimized for a ^2,3^*J* of 8 Hz. Mass spectra were obtained on a LTQ OrbitrapXL (Thermo Scientific) mass spectrometer. Medium pressure liquid chromatography was performed on a Büchi apparatus using a silica gel (230–400 mesh) column; HPLC were achieved on a Knauer apparatus equipped with a refractive index detector. The Knauer HPLC apparatus was used to purify and assess purity (>95%) of all final products. LUNA (Phenomenex) columns (reverse phase, RP18, or normal phase, SI60, 250 × 4 mm) were used.

### 3.2. Animal Material, Extraction and Isolation

Specimens of *Leucetta* sp. (310 g wet weight) were collected in January 2010 in the Bunaken Marine Park of Manado along the coasts of the small island of Siladen (North Sulawesi, Indonesia) at a depth of 2–5 m. A voucher sample (MAN-10-08) was deposited at the Dipartimento di Scienze del Mare, Università Politecnica delle Marche. 

The sponge was repeatedly extracted with MeOH and CHCl_3_ at room temperature and the obtained combined material (8.6 g) was partitioned between H_2_O and EtOAc to give an acetate extract (0.45 g), while the water phase was further partitioned against *n*-BuOH, thus affording a butanol extract (1.2 g). This was chromatographed by MPLC over silica gel (230–400 mesh) eluting with a gradient system of increasing polarity from EtOAc to MeOH. Fractions eluted with EtOAc/MeOH 7:3 were further purified by RP18 HPLC (MeOH/H_2_O 55:45) to afford leucettamols A (**1**, 452.0 mg) and B (**3**, 45.5 mg) in the pure state. 

### 3.3. Acetylation of Leucettamols

Leucettamol A (**1**, 15.0 mg, 0.032 mmol) was dissolved in dry pyridine (1.5 mL) and treated with Ac_2_O (1.5 mL). After standing overnight under stirring at room temp, the reaction was worked up by addition of a few drops methanol to destroy the excess Ac_2_O, water (*ca.* 1 mL) and EtOAc (*ca.* 3 mL). The organic phase was washed sequentially with 2 N H_2_SO_4_, sat. NaHCO_3_ and brine. After drying (Na_2_SO_4_) and removal of the solvent, the residue was purified by HPLC (*n*-hexane/EtOAc 85:15) to afford 13.5 mg (66% yield) of the tetra-acetate **2**, identified by comparison with data reported in [30]. When leucettamol B (**3**, 5.0 mg, 0.010 mmol) was subjected to the same reaction procedure, 4.5 mg (64% yield) of the penta-acetylated derivative **4** were obtained. Compound **4**: ^1^H NMR (500 MHz, CDCl_3_): δ 6.20 (1H, dd, *J* = 15.0 and 11.0 Hz, *H*-10), 5.80 (1H, dd, *J* = 15.0 and 6.0 Hz, *H*-9), 5.45–5.40 (10H, overlapped, *H*-5, *H*-6, *H*-11, *H*-12, *H*-14, *H*-15, *H*-17, *H*-18, *H*-20, *H*-21), 4.93 (1H, m, *H*-8), 4.83 (2H, m, *H*-3 and *H*-28), 4.18 (2H, m, *H*-2 and *H*-29), 2.80 (4H, m, *H*_2_-4 and *H*_2_-7), 2.75 (6H, m, *H*_2_-13, *H*_2_-16 and *H*_2_-19), 2.10–2.05 (15H, s, 5 × *Ac*), 2.04 (2H, m, *H*_2_-22), 1.50–1.40 (10H, m, *H*_2_-23 to *H*_2_-27), 1.16 (6H, d, *J* = 6.9 Hz, *Me*-1 and *Me*-30). ESIMS (positive ions): *m/z* 721 [M + Na]^+^.

### 3.4. Reduction of Leucettamol A and Acetylation of Compound **5**

Leucettamol A (**1**, 34.0 mg) was treated with palladium-charcoal in EtOH (6 mL) under a hydrogen atmosphere at room temperature for 18 h. After filtration of the catalyst, the solvent was evaporated and the residue was purified by RP18 HPLC (MeOH/H_2_O 7:3) to give the saturated compound **5** (36.5 mg, 100%), whose spectroscopic data were identical with those reported in [30]. Compound **5 **(8.0 mg, 0.016 mmol) was subjected to acetylation following the same procedure described below and gave compound **6** (10.5 mg) in quantitative yields. Compound **6**: ^1^H NMR (500 MHz, CDCl_3_): δ 4.82 (2H, m, *H*-3 and *H*-28), 4.16 (2H, m, *H*-2 and *H*-29), 2.09 (6H, s, *OAc*), 1.96 (6H, s, *OAc*), 1.58–1.48 (4H, m, *H*_2_-4 to *H*_2_-27), 1.25–1.20 (42H, *H*_2_-5 to *H*_2_-26), 1.15 (6H, d, *J* = 6.9 Hz, *Me*-1 and *Me*-30). ESIMS (positive ions): *m/z* 675 [M + Na]^+^. 

### 3.5. *In Vitro* Assays with TRP Receptors

Assays of TRP-mediated elevation of intracellular [Ca^2+^] were performed as previously described [40]. In the current study we have used wild-type HEK293 cells, cells stably expressing rat TRPA1 or human TRPV1 or rat TRPM8. HEK-293 cells stably over-expressing recombinant rat TRPA1, rat TRPM8 or human TRPV1 were selected by G-418 (Geneticin; 600 μg·mL^−1^), grown on 100 mm diameter Petri dishes as monolayers in minimum essential medium supplemented with non-essential amino acids, 10% fetal bovine serum and 2 mM glutamine, and maintained under 5% CO_2_ at 37 °C. Stable expression of each channel was confirmed by real time quantitative PCR (not shown) [32,41,42]. On the day of the experiment, the cells were loaded for 1 h at 25 °C with the cytoplasmic calcium indicator Fluo-4AM (Invitrogen Carlsbad, CA, USA) 4 μM in DMSO containing 0.02% Pluronic F-127 (Invitrogen, Carlsbad, CA, USA). After loading, cells were washed twice in Tyrode’s buffer (145 mM NaCl, 2.5 mM KCl, 1.5 mM CaCl_2_, 1.2 mM MgCl_2_, 10 mM D-glucose and 10 mM HEPES, pH 7.4), re-suspended in the same buffer, and transferred to the quartz cuvette of the spectrofluorimeter (λ_ex_ = 488 nm; λ_em_ = 516 nm) (Perkin-Elmer LS50B equipped with PTP-1 Fluorescence Peltier System; Perkin-Elmer Life and Analytical Sciences, Waltham, MA, USA) under continuous stirring. About 100,000 cells were used for each data point. Experiments were carried out at 22 °C by measuring cell fluorescence before and after the addition of various concentrations of test compounds. The values of the effect on [Ca^2+^]_i_ in wild-type (*i.e.*, not transfected with any construct) HEK-293 cells were taken as baselines and subtracted from the values obtained from transfected cells. The potency of test compounds (EC_50_ values) was determined as the concentration of test substances required to produce half-maximal increases in [Ca^2+^]_i_. The efficacy of the agonists on TRPA1 is expressed as a percentage of the effect obtained with 100 μM allylisothiocyanate (AITC). Antagonist/desensitizing behaviour was evaluated against icilin (0.25 μM) for TRPM8 and AITC (100 μM) for TRPA1 by adding the compounds in the quartz cuvette 5 min before stimulation of cells with agonists. Data are expressed as the concentration exerting a half-maximal inhibition of agonist effect (IC_50_). All determinations were at least performed in triplicate. Dose-response curves were fitted by a sigmoidal regression with variable slope. Curve fitting and parameter estimation were performed with GraphPad Prism (GraphPad Software Inc., San Diego, CA, USA).

### 3.6. CB_1_ and CB_2_ Receptor Binding Assays

Membranes harvested from human recombinant CB_1_ (B_max_ = 2.5 pmol/mg protein) or CB_2_ (B_max_ = 4.7 pmol/mg protein) receptor-transfected HEK-293 cells were incubated with the high-affinity ligand [^3^H]-CP-55,940 (0.14 nM, *K*_d_ = 0.18 or 0.084 nM, *K*_d_ = 0.31 nM, respectively, for CB_1_ and CB_2_), and displaced with 10 μM of the heterologous competitor for nonspecific binding, WIN-55212-2 (*K*_i_ values 9.2 and 2.1 nM, respectively, for CB_1_ and CB_2_). All compounds were assayed according to the manufacturer’s (Perkin-Elmer, Milano, Italy) instructions. Increasing concentrations of compounds were incubated with [^3^H]-CP-55,940 for 90 min at 30 °C to generate displacement curves. IC_50_ values of the test compounds for the displacement of the bound radioligand were obtained by GraphPad Prism and used to calculate *K*_i_ values via the Cheng-Prusoff equation. Data are represented as means of at least *n* = 3 experiments. 

## 4. Conclusions

The current knowledge of TRP channels is strongly indebted to the basic research in the field of natural products chemistry and the biological evaluation of several naturally occurring substances, especially from plants, has been a driving force for the characterization of many TRP channels. For example, attempts to understand the hot and painful action of capsaicin and of its ultrapotent analogue resiniferatoxin, led to the cloning of the vanilloid channel TRPV1 [5]. More recently, two additional TRP channels, TRPM8 and TRPA1, have been identified as novel and potentially useful targets for the treatment of several pathological conditions and, they currently represent a hot topic of the research in this field.

In this paper we have reported that leucettamols, bifunctionalized sphingoid-like compounds of marine origin, act as activators of the TRPA1 channel and inhibitors of the icilin-mediated activation of the TRPM8 channel. To our knowledge, leucettamols represent the first compounds of marine origin to target TRPA1 and/or TRPM8 channels. Although leucettamols bear a structural resemblance with anandamide, including also the *cis *double bond system required to adopt the bent U-shaped conformation, they proved to be completely inactive at CB_1_ and CB_2_ receptors. They were also inactive toward TRPV1 channels (only leucettamol B showed a very modest activity). 

Leucettamols belong to a somewhat rare class of natural products and, to date, only a handful of two-headed sphingoid-like compounds have been found in nature, mostly from marine sponges, including rhizochalins [43,44,45,46], ocenapiside [47], and calyxoside [48]. These compounds differ from leucettamols in the length/functionalization of the chain and/or in the configuration at the stereogenic centers and it would be very interesting to test them on TRP channels. Moreover, the relatively simple structure of leucettamols, especially that of the fully saturated semi-synthetic analogue **5**, should guarantee the feasibility of a total chemical synthesis, thus allowing a more detailed evaluation of this new class of TRP ligands as a possible approach for the treatment of neuropathic and inflammatory pain and of prostate cancer.
